# Molecular characterization of *Echinococcus* species causing human cystic echinococcosis in Algeria: predominance of *Echinococcus granulosus* sensu stricto

**DOI:** 10.1051/parasite/2026058

**Published:** 2026-10-08

**Authors:** Rania Sihem Boussa, Perrine Duquet, Coralie Barrera, Elise Roy, Manel Amri, Imene Soufli, Houda Belguendouz, Nassim Sid-Idris, Chafia Touil-Boukoffa, Laurence Millon, Karima Achour, Jenny Knapp

**Affiliations:** 1 Team Cytokines and NOSynthases: Immunity and Pathogeny, LBCM; FSB, USTHB Algiers Algeria; 2 National Reference Centre for Echinococcoses, Department of Parasitology-Mycology University Hospital of Besançon Besançon France; 3 Chrono-Environnement Laboratory UMR/CNRS 6249, Université Marie et Louis Pasteur, Université Marie et Louis Pasteur Besançon France; 4 Department of Surgery, Djillali Belkhenchir Hospital Algiers Algeria; 5 University of Health Sciences Youcef El Khatib Algiers Algeria; 6 Algerian Academy of Sciences and Technologies Algiers Algeria; 7 Department of Thoracic Surgery, University Hospital Center Lamine Debaghine Algiers Algeria

**Keywords:** Cystic echinococcosis, Genotyping, *Echinococcus granulosus* sensu stricto, *Echinococcus canadensis*, Algeria

## Abstract

Genotyping of cystic echinococcosis (CE) specimens enables the identification of predominant species and genotypes, yielding critical insights into disease epidemiology. Species identification is essential for the development of effective prevention and control strategies. In this context, we assessed the molecular diversity of *E. granulosus* sensu lato among surgically confirmed human CE cases recruited from Algerian referral hospitals. Hydatid cysts were collected from 31 patients undergoing surgery for pulmonary and/or hepatic CE in Algeria between 2022 and 2024. Hydatid fluid, cyst wall, and laminated-layer preparations were used for DNA extraction. End-point PCR coupled with Sanger sequencing, targeting the mitochondrial 12S-*rrnS*, *nad5*, and *nad2* markers, was performed for species identification and genotyping, with real-time PCR (qPCR) performed in parallel. End-point PCR was successful in 29 cases (93.5%), while qPCR detected DNA of *Echinococcus* spp. in 30 cases (96.8%). The additional qPCR-positive sample showed late amplification but could not be assigned to a genotype because no sequence was obtained. Among the 29 molecularly characterized samples, *E. granulosus* sensu stricto genotype G1 was predominant and was identified in 27 samples (93.1%). One isolate was identified as *E. granulosus* sensu stricto genotype G3, and one as *E. canadensis* G6/G7. Genotype G1 and G3 cases were distributed in northern and central Algeria, while the G6/G7 isolate was detected in a patient from Tindouf, southern Algeria. These results demonstrate the predominance of *E. granulosus* sensu stricto genotype G1 among the molecularly characterized human cystic echinococcosis cases in this series.

## Abstract (fr)

Le génotypage d'échantillons d'échinococcose kystique (EK) permet d'identifier les espèces et génotypes prédominants, apportant ainsi des informations cruciales sur l'épidémiologie de la maladie. L'identification des espèces est essentielle pour élaborer des stratégies efficaces de prévention et de lutte. Dans ce contexte, cette étude a évalué la diversité moléculaire d'*E. granulosus* sensu lato chez des patients atteints d'EK confirmée chirurgicalement, recrutés dans des hôpitaux de référence en Algérie. Des kystes hydatiques ont été prélevés chez 31 patients opérés pour une EK pulmonaire et/ou hépatique en Algérie entre 2022 et 2024. Le liquide hydatique, la paroi du kyste et des préparations de la membrane lamellaire ont été utilisés pour l'extraction de l'ADN. Une PCR en point final couplée au séquençage Sanger, ciblant les marqueurs mitochondriaux 12S-*rrnS*, *nad5* et *nad2*, a été réalisée pour l'identification des espèces et le génotypage, parallèlement à une PCR en temps réel (qPCR). La PCR en point final a donné un résultat positif dans 29 cas (93,5 %), tandis que la qPCR a détecté de l'ADN d'*Echinococcus* spp. dans 30 cas (96,8 %). L'échantillon supplémentaire positif en qPCR a présenté une amplification tardive mais n'a pu être attribué à un génotype, aucune séquence n'ayant été obtenue. Parmi les 29 échantillons caractérisés sur le plan moléculaire, le génotype G1 d'*E. granulosus* sensu stricto était prédominant et a été identifié dans 27 échantillons (93,1 %). Un isolat a été identifié comme appartenant au génotype G3 d' *E. granulosus* sensu stricto, et un autre comme *E. canadensis* G6/G7. Les cas associés aux génotypes G1 et G3 étaient répartis dans le nord et le centre de l'Algérie, tandis que l'isolat G6/G7 a été détecté chez un patient originaire de Tindouf, dans le sud du pays. Ces résultats démontrent la prédominance du génotype G1 d'*E. granulosus* sensu stricto parmi les cas d'échinococcose kystique humaine caractérisés moléculairement dans cette série.

## Introduction

Parasitic food diseases, such as echinococcosis, represent a major public health issue. Recent global burden analyses estimated that cystic echinococcosis accounted for about 148,521 incident cases, 633,404 prevalent cases, 1,364 deaths, and 105,072 disability-adjusted life years (DALYs) globally in 2021 [49]. Cystic echinococcosis (CE) is a disease of global importance, particularly affecting North Africa, Central Asia, and South America [53]. This zoonosis causes serious medical complications and has a significant socio-economic impact, highlighting the urgency of strengthening prevention and control. However, in the absence of further progress in prevention and control strategies, the number of cases will continue to increase [7, 54].

CE is a zoonotic disease caused by cestodes belonging to the *Echinococcus granulosus* sensu lato (s.l.) complex. Its life cycle involves carnivores, mainly dogs, as definitive hosts (DHs), and domestic ungulates, particularly sheep, as intermediate hosts (IHs). Humans are accidental intermediate hosts. In intermediate hosts, the parasite develops hydatid cysts in various organs. The liver is the most frequently affected organ, in approximately 74.2% of cases, followed by the lungs in 24.2% [52]. The cyst is composed of a fluid-filled vesicle, hydatid fluid containing protoscoleces, and daughter vesicles, all surrounded by three layers, characterized as host-derived collagenous layer, an outer acellular laminated layer, and a germinal layer. This trilayer structure plays a crucial role in the function of the cyst and interaction with the host [21, 50].

This zoonosis is caused by species from the *E. granulosus* s.l. complex*,* presenting high genetic diversity. According to the current taxonomy, *E. granulosus* s.l. includes *E. granulosus* sensu stricto (s.s.) (G1 and G3), *E. equinus* (G4), *E. ortleppi* (G5), *E. canadensis* (G6/G7 and G8/G10), and *E. felidis* [50]. Genotype G2 is no longer considered a valid genotype and is currently regarded as a microvariant of G3, based on recent phylogenetic evidence [10, 28]. These taxa differ in their geographical distribution, host specificity, and transmission patterns. The domestic life cycle of *E. granulosus* s.s. mainly involves dogs as definitive hosts and sheep as intermediate hosts, although a wide range of other livestock species may also serve as intermediate hosts. Of note, *E. canadensis* G6 is mainly associated with a camel-dog cycle, whereas G7 is more commonly associated with a pig-dog cycle; nevertheless, other domestic intermediate hosts may contribute to their circulation [10]. Among these taxa, *E. granulosus* s.s. and *E. canadensis* are the principal causative agents of human CE [2, 10]. Consequently, molecular characterization is essential for accurately identifying circulating species and genotypes, improving our understanding of parasite diversity, and elucidating transmission dynamics [2, 14, 50].

In Algeria, CE is highly endemic, with infections reported in dogs, livestock, and humans [6, 23, 52, 55]. Studies conducted in slaughterhouses in central and eastern Algeria reported prevalence rates of *E. granulosus* s.l. infection in ruminants ranging from 4.9% to 10%. Notably, cattle exhibited higher infestation rates than sheep, although cyst fertility was lower in cattle [16, 27].

A combination of socio-economic and environmental factors drives transmission of the parasite. Previous molecular studies conducted in Algeria have shown that *E. granulosus* s.s., particularly genotype G1, is the predominant species infecting both humans and livestock, although genotype G3 and *E. canadensis* (G6) have also been reported [5, 44, 55]. Despite the endemicity of the disease, data on the epidemiology and genetic diversity of Algerian genotypes remain limited. Karshima *et al.* [26] highlighted the scarcity of molecular epidemiological data from human CE cases in Algeria and emphasized the need for additional studies. A recent study by Nocerino and co-workers revealed high *E. granulosus* prevalence in animals in Ouargla region (>50%) and moderate prevalence in Adrar region (5.01–25%), located in central and northeastern Algeria. However, according to their study, a large part of the territory remains unstudied [41]. Similarly, original epidemiologic investigations have mainly been conducted in a limited number of regions and have focused predominantly on livestock, leaving several geographical areas and host species insufficiently investigated [1, 23]. It is therefore essential to further characterize the circulating *Echinococcus* species and genotypes in different regions of Algeria in order to improve our understanding of CE epidemiology and transmission dynamics.

The aim of this study was to identify the species and genotypes of *E. granulosus* s.l. responsible for CE in Algerian patients using advanced molecular tools, in order to contribute to the epidemiologic knowledge on the disease in Algeria and to improve prevention and control strategies. In this context, we evaluated the effectiveness of the molecular diagnostic methods used, such as end-point PCR, Sanger sequencing, and quantitative real time PCR (qPCR) for the discrimination of species and genotypes. Moreover, epidemiologic data concerning the patients with CE were collected and analyzed.

## Patients and methods

### Ethics

This study was conducted in accordance with the guidelines of the local Ethics Committee. Informed consent was obtained from all participants before their inclusion in the study.

### Patients

A total of 31 patients diagnosed with pulmonary or hepatic CE, or both, were included in this study. All patients were admitted to the Department of Thoracic Surgery at the Lamine Debaghine Hospital, and the Department of Surgery at the Djilali Belkhenchir Hospital (Algiers, Algeria) from 2022 to 2024. Only one cyst was analyzed per patient. In patients presenting both pulmonary and hepatic localizations, the pulmonary cyst was selected for molecular analysis because these patients were recruited through the Department of Thoracic Surgery. Hepatic cysts from these patients were not surgically sampled within this department and were therefore unavailable for molecular characterization. The patients came from different localities in Algeria, mainly from Algiers. The mean age was 42 ± 18.1 years and none of the patients had other parasitic infections. Clinical and epidemiologic data are presented in Table 1.

**Table 1:** Clinical and pathology data from patients diagnosed with hydatid cysts. The size corresponds to the hydatid cyst that was collected and analyzed for each patient.

Characteristics	Patients *n* = 31
Age group (years) *n* (%)	
<20	4 (12.9%)
20–39	10 (32.3%)
40–59	10 (32.3%)
≥60	7 (22.6%)
Sex (female/male)	15/16

Lesion location *n* (%)		
Lung	19 (61.3%)	
Liver	7 (22.6%)	
Lung + Liver	5 (16.1%)	
Hydatid cyst size n (%)		
<50 mm	8 (25.81%)	
50–100 mm	17 (54.84%)	
> 100 mm	6 (19.35%)	

**Figure 1:**
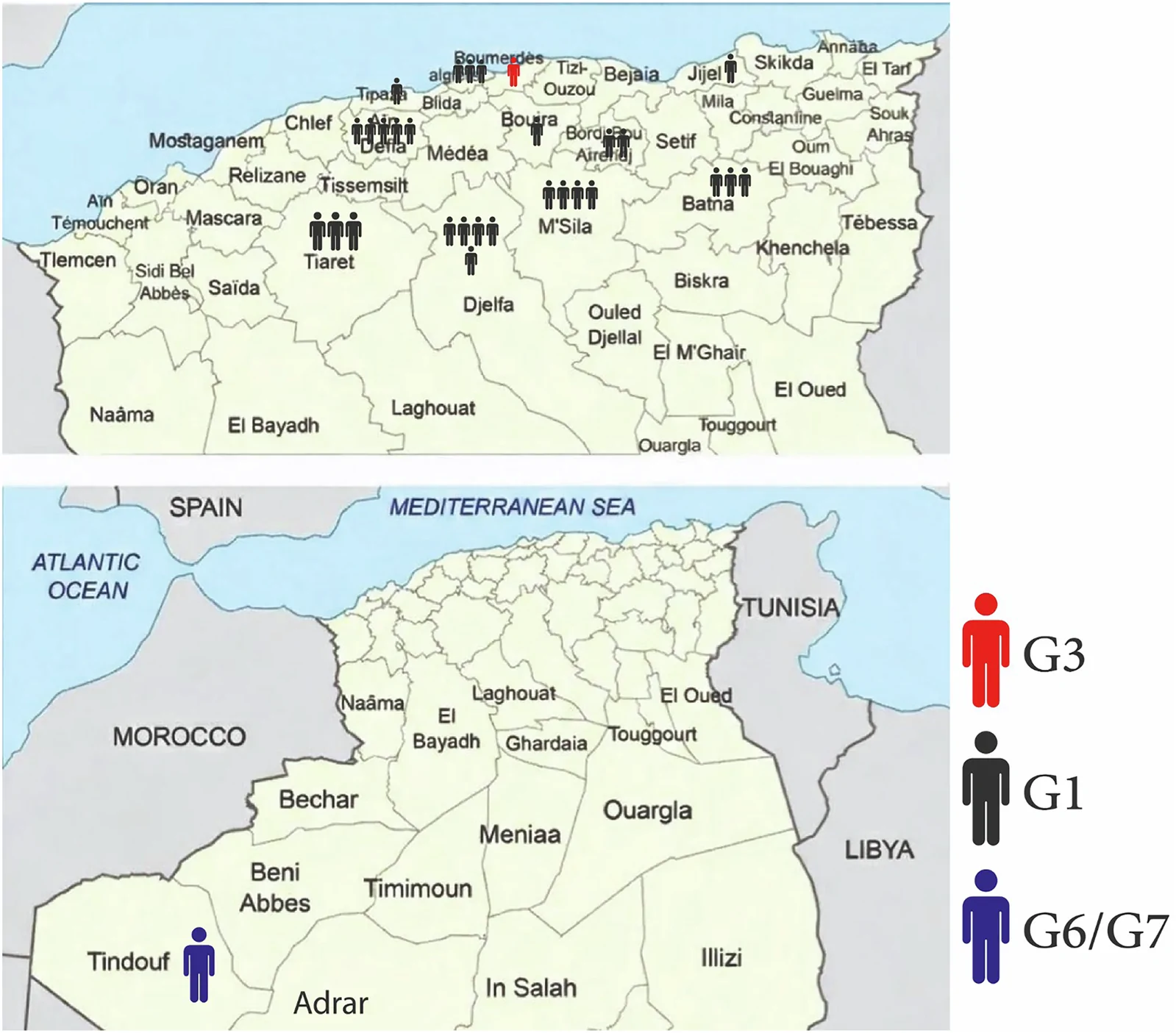
Geographic distribution of *Echinococcus granulosus* sensu lato genotypes across Algerian provinces.

### Preparation of specimens from hydatid cysts

Hydatid fluid samples were first collected under sterile conditions. The collected fluids were then examined microscopically for the presence of protoscoleces in the Algerian laboratory. From these observations, six hydatid fluid samples containing protoscoleces were included in this study. Additionally, 25 cyst walls were recovered and thoroughly washed. Among these, seven cyst walls underwent further separation to isolate the laminated layer (Table 3). All samples were preserved at −20 °C prior to laboratory processing.

### DNA extraction

DNA was extracted from hydatid samples at the laboratory of Parasitology-Mycology, University Hospital of Besançon*,* France, including hydatid fluid and/or cyst walls and laminated layer, using a High Pure PCR Template Preparation Kit (Roche Diagnostics, Mannheim, Germany), following the manufacturer’s instructions. The purified DNA was eluted in 200 μL of the provided buffer. The concentration of DNA was measured using 2 μL of each sample with a nanophotometer apparatus (Implen, Munich, Germany) (Table 3), in order to control a minimum DNA concentration for the molecular analysis. DNA samples were then stored at −20 °C until use.

### Species identification and genotyping

The first step of species identification was performed using a mitochondrial target corresponding to part of the 12S-rRNA gene, targeting a 267-bp fragment [43]. The designed degenerate primers allow the amplification of all *Echinococcus* species, as well as some *Taenia* species. Species identification was achieved by Sanger sequencing of the amplicons, followed by comparison with sequences available in the National Center for Biotechnology Information (NCBI) nucleotide database. In the second step, which was genotyping of the *E. granulosus* s.l. complex, two other mitochondrial targets were selected: part of the *nad5* gene, targeting a 759 bp fragment, to differentiate *E. granulosus* s.s. genotypes G1 from G3, and the *nad2* gene, targeting a 781 bp fragment, to differentiate *E. canadensis* genotype G6 from G7 [29, 36]. End-point PCR reactions were conducted in a final volume of 20 μL, containing 10 μL of 2X AmpliTaq Gold 360 master mix (Applied Biosystems, Vilnius, Lithuania), 0.5 μM final concentration of each forward and reverse primer, and 1 μL of genomic DNA. Primers and PCR conditions followed literature recommendations (Table 2). PCR amplification was performed in a Biometra T3 thermocycler (Whatman Biometra, Göttingen, Germany). An *E. granulosus* s.s. G1 positive control and a negative control (H_2_O) were included in each PCR run. Inhibition controls were conducted in the PCR 12S-*rrn*S by mixing 1 μL of *Taenia saginata* extract DNA. Amplicons were visualized on 1.5% (w/v) agarose gels stained with 10% SYBR Safe DNA Gel Stain (Invitrogen, Carlsbad, CA, USA), in 1X tris-acetate EDTA (TAE) buffer, with the expected size products (Table 2).

**Table 2:** Molecular targets tested, PCR conditions, and references.

Target species	Target gene	Primer	5′–3′ sequence	Label (qPCR probes)	PCR product size (bp)	PCR program	Reference
*Echinococcus* spp.	*12S-rrnS* rRNA	12S-Cest3-Fwd	YGAYTCTTTTTAGGGGAAGGTGTG		267	Pre-denat.: 10 min/94 °C, 40 cycles: 30 sec/94 °C, 30 sec/55 °C, 60 sec/72 °C, Final elong.: 2 min/72 °C	Roelfsema *et al.* [43]
		12S-Cest4-Rev	GCGGTGTGTACMTGAGCTAAAC				
*Echinococcus granulosus* s.s. genotypes G1-G3	*nad5*	EG-G1G3-nd5-F1	GTTGTTGAAGTTGATTGTTTTGTTTG		759	Pre-denat.: 10 min/95 °C, 30 cycles: 20 sec/95 °C, 45 sec/52.5 °C, 60 sec/72 °C, Final elong.: 5 min/72 °C	Kinkar *et al.* [29]
		EG-G1G3-nd5-R1	GGAACACCGGACAAACCAAGAA				
*Echinococcus canadensis genotypes G6-G7*	*nad2*	EC-G7for	GTGTTGTGTTGTTGATAGATTG		781		Laurimäe *et al.* [36]
		EC-G7rev	GTAAAAATAATCACCACCCAAC				
*Echinococcus granulosus* s.s.	*cox3*	Eg_cox3_Fwd_147	TATCTGTAACACCACAAAACTCAAACC		147	Pre-denat:120 sec/50 °C, 10 min/95 °C, 45 cycles: 15 sec/95 °C, 60 sec/60 °C (ramp: 1.6 °C/sec)	Knapp *et al.* [32]
		Eg_cox3_Rev_147	CGTTGGAGATTCCGTTTGTTG				
		Eg_cox3_Probe_147	AACAAAAGCAAATCACAACAACGTCAACCC	3'CY5-5'BHQ2			
*Echinococcus canadensis*	*nad5*	Ec_nad5_Fwd_105	ATACGCCATGACTTATCAACTGAAAT		105		Knapp *et al.* [32]
		Ec_nad5_Rev_105	AGATTGTGGCTTTGTCAACTTGTAA				
		Ec_nad5_Probe_105	TCACCAAAAATCAAGTACAAAACGCACCAA	3'FAM-5'TAMRA			
Alea inhibitor control	None	Alea_Fwd_127	CCTAAAAATGTCTATGATTGGTCCACTA		101		
		Alea_Rev_228	GGGAGTACCTTGCCATACAAAATT				
		Alea_probe_162	TTAAATCAACTCCTAAATCCGCGCGATAGG	3'VIC-5'TAMRA			Knapp *et al.* [31]

Positive PCR products on electrophoresis were subjected to Sanger sequencing using the Big Dye Terminator v3.1 reagent (Applied Biosystems), according to the manufacturer’s protocol, with the same primers used in the initial amplification step. Sequencing was performed on a 3500xL Dx Genetic Analyzer (Applied Biosystems). Chromatograms from forward and reverse sequences were aligned and manually edited using BioEdit software v7.2.6 [22]. Nucleotide sequences (part of the 12S-*rrn*S, *nad2*, and *nad5* genes) were compared using the BLAST program from the NCBI database.

### Real-Time Quantitative PCR

Real-time quantitative PCR (qPCR) was performed as a second-line molecular assay to evaluate its performance for species identification, particularly in samples with low parasite DNA content or those that were negative by conventional PCR. This multiplex assay detects species from the *E. granulosus* s.l. complex [32], according to the specific primer and probe sets used (*E. granulosus* s.s. and *E. canadensis* for this study). A duplex qPCR was performed in order to monitor PCR inhibitors with parasite detection simultaneously, in a final volume of 20 μL, containing 10 μL of 2X TaqMan Gene Expression master mix (Life Technologies, Foster City, CA, USA), 0.5 μM of each primer, 0.02 μM of hydrolysis probes final concentration with compatible fluorochromes (Table 2), and 1 μL of total DNA extracted from the specimens. The reaction was run on a QuantStudio 5 Real-Time PCR System (Life Technologies) for 45 cycles [32] (Table 2). Each sample was tested in duplicate, and Cq values were recorded for both replicates. A qPCR result for parasite detection was considered positive if at least one duplicate showed a Cq value <45 cycles. PCR inhibitors were monitored using the internal Alea qPCR control (Table 2) and amplified in a duplex qPCR [31]. For the detection of PCR inhibition, samples with a Cq value of 35–45 cycles were considered partially inhibited, whereas those with a Cq value >45 cycles were considered fully-inhibited. When an amplification was obtained with the *Echinococcus* targeted qPCR (Cq >45) and inhibition was detected, the DNA was diluted 1:10 and the PCR repeated. The qPCR results were analyzed using Design Analysis software v2.5 (Applied Biosystems).

## Results

For the identification of *E. granulosus* s.l. genotypes in cases of concomitant pulmonary and hepatic cysts, genotyping was performed on the pulmonary cyst only. Molecular analysis for species identification was performed on the 31 hydatid cyst samples, using a partial sequence of the mitochondrial 12S-*rrnS* gene. Among these samples, 29 (93.5%) yielded positive 12S-*rrn*S PCR amplification, while 2 (6.5%) remained negative (Table 3). A12 and A20 remained negative despite the use of optimized PCR conditions (Table 3). A12 corresponded to cyst-wall material, whereas A20 corresponded to a laminated-layer preparation.

**Table 3:** Molecular characterization and quantitative real time PCR results of *Echinococcus granulosus* sensu lato isolates from human cystic echinococcosis cases. For double localization (lungs+liver), genotyping was performed on lung samples.

Sample ID	Species	Genotype	Specimen	DNA concentration (ng/μL)	OD ratio 260/280	Organ	Location	Occupation	qPCR EgG1 (1)	qPCR EgG1 (2)	qPCR EgG6/7 (1)	qPCR EgG6/7 (2)	qPCR Alea (mean)
A1	*E. granulosus* s.s.	G1	Hydatid fluid	6.2	2.08	liver	Algiers	Unemployed	34.9	34.8	>45	>45	33.0
A2	*E. granulosus* s.s.	G1	Laminated layer	5.7	1.6	liver	Djelfa	Housewife	27.2	27.2	>45	>45	34.3
A3	*E. granulosus* s.s.	G1	Cyst wall	21.2	1.9	lungs	Algiers	Self-employed	27.1	27.2	>45	>45	31.8
A4	*E. granulosus* s.s.	G1	Hydatid fluid	2.3	2.5	lungs	Tipaza	Housewife	27.7	27.5	>45	>45	33.3
A5	*E. granulosus* s.s.	G1	Hydatid fluid	9.4	1.7	lungs	Bordj Bou Arreridj	Unemployed	27.9	27.8	>45	>45	31.3
A6	*E. granulosus* s.s.	G1	Cyst wall	1.5	2.1	lungs	Tiaret	Housewife	30.1	30.3	>45	>45	33.3
A7	*E. granulosus* s.s.	G1	Laminated layer	6.9	2.0	liver	Tiaret	Unemployed	28.5	28.4	>45	>45	31.0
A8	*E. canadensis*	G6/G7	Cyst wall	6.0	1.2	liver	Tindouf	Nurse	>45	>45	29.2	29.2	31.4
A9	*E. granulosus* s.s.	G1	Hydatid fluid	1.8	0.8	lungs	Djelfa	Housewife	23.4	23.5	>45	>45	35.4
A10	*E. granulosus* s.s.	G1	Cyst wall	14.6	1.3	lungs+liver	Msila	Housewife	22.3	22.1	>45	>45	34.7
A11	*E. granulosus* s.s.	G1	Cyst wall	4.0	1.0	lungs+liver	Boussada	Farmer	23.0	22.9	>45	>45	33.6
A12	-	-	Cyst wall	32.8	1.8	lungs	Tiaret	Housewife	43.5	>45	>45	>45	31.7
A13	*E. granulosus* s.s.	G1	Cyst wall	8.5	1.5	lungs	Bordj Bou Arreridj	Laborer	29.9	29.8	>45	>45	33.4
A14	*E. granulosus* s.s.	G1	Laminated layer	5.5	1.8	liver	Khemis Miliana	Driver	29.4	29.4	>45	>45	34.1
A15	*E. granulosus* s.s.	G1	Cyst wall	13.5	1.5	lungs+liver	Ain Defla	Housewife	33.9	33.8	>45	>45	33.2
A16	*E. granulosus* s.s.	G1	Cyst wall	3.4	0.9	lungs	Msila	Unemployed	30.3	30.2	>45	>45	35.3
A17	*E. granulosus* s.s.	G1	Cyst wall	4.9	1.4	liver	Jijel	Housewife	26.9	26.9	>45	>45	32.2
A18	*E. granulosus* s.s.	G1	Cyst wall	98.1	1.8	lungs+liver	Ain Defla	Housewife	32.9	32.7	>45	>45	34.7
A19	*E. granulosus* s.s.	G3	Cyst wall	17.9	1.8	lungs+liver	Boumerdes	Production worker	31.5	31.2	>45	>45	34.0
A20	-	–	Laminated layer	ND	ND	lungs	Algiers	Unemployed	>45	>45	>45	>45	31.5
A21	*E. granulosus* s.s.	G1	Laminated layer	2.5	1.5	lungs	Batna	Housewife	27.5	27.6	>45	>45	31.9
A22	*E. granulosus* s.s.	G1	Cyst wall	3.5	1.6	lungs	Msila	Legal assistant	28.8	28.9	>45	>45	34.6
A23	*E. granulosus* s.s.	G1	Cyst wall	11.0	1.7	lungs	Batna	Unemployed	44.1	43.4	>45	>45	31.7
A24	*E. granulosus* s.s.	G1	Cyst wall	35.7	1.8	lungs	Khemis Miliana	Driver	24.4	24.5	>45	>45	31.1
A25	*E. granulosus* s.s.	G1	Cyst wall	6.1	1.6	lungs	Tiaret	Farmer	31.4	31.7	>45	>45	33.6
A26	*E. granulosus* s.s.	G1	Laminated layer	6.4	1.7	lungs	Msila	Unemployed	24.7	24.4	>45	>45	33.0
A27	*E. granulosus* s.s.	G1	Cyst wall	7.7	1.6	lungs	Algiers	Nurse	28.9	29.0	>45	>45	31.5
A28	*E. granulosus* s.s.	G1	Laminated layer	9.6	2.1	lungs	Khemis Miliana	Laborer	25.1	25.1	>45	>45	29.3
A29	*E. granulosus* s.s.	G1	Hydatid fluid	5.6	1.7	lungs	Batna	Housewife	24.9	25.2	>45	>45	32.9
A30	*E. granulosus* s.s.	G1	Hydatid fluid	13.9	1.4	lungs	Ain Defla	Unemployed	37.1	37.8	>45	>45	32.0
A31	*E. granulosus* s.s.	G1	Cyst wall	14.3	1.9	liver	Djelfa	Housewife	21.9	22.0	>45	>45	32.5

**Table 4:** Literature review of molecular studies on *Echinococcus granulosus* sensu lato in human (*n* = 418) and livestock hosts in Algeria.

Host species	Geographic area	Cysts molecularly analyzed	Molecular markers	Identified species/genotypes and main findings	Study
Humans, ovine, cattle and dromedaries	Mainly eastern Algeria, including Touggourt; additional comparative samples from other African countries	46 Algerian cysts; 51 cysts in total	Partial *cox*1, *nad*1 and nuclear BG1/3 target	*E. granulosus* s.s. G1 in Algerian humans, sheep and cattle; camel-associated G6 in Algerian dromedaries	Bardonnet *et al.* [4]
Humans, sheep, cattle and dromedaries	Northern Algeria and Touggourt–Ouargla; multicountry study also including Mauritania and Ethiopia	46 Algerian cysts; 71 cysts in total	*cox*1, *nad*1, *act*II and *hbx*2	G1, historically assigned G2, and G6; G6 mainly detected in dromedaries	Maillard *et al.* [37]
Humans, sheep, cattle, goats and dromedaries	Human cases mainly recruited in Algiers; livestock from Tiaret and Tamanrasset	70 cysts: 54 human and 16 livestock cysts	*cox*1 and *nad*1	Human isolates: G1, n = 49; G3, *n* = 4; G6, *n* = 1. Livestock isolates: G1 in sheep, cattle and goats; G6 in two dromedaries	Zait *et al.* [55]
Sheep and cattle	Djelfa Province	125 cysts	Near-complete mitochondrial *cox*1 sequence, 1,609 bp	All isolates belonged to *E. granulosus* s.s.; 73 *cox1* haplotypes were identified	Laatamna *et al.* [33]
Humans	Western Algeria	46 cysts	Partial *cox*1a, *cox1b*, *nad*3 and *atp*6 sequences	All isolates belonged to *E. granulosus* s.s. genotype G1; 19 concatenated haplotypes were identified	Moussa *et al.* [39]
Dromedaries	Tindouf and Illizi	75 cysts from 49 dromedaries	*nad*5 and *nad*2	G1, G3 and G6; 11 haplotypes, including six G6 haplotypes	Samari *et al.* [44]
Sheep, cattle and dromedaries	Six slaughterhouses in eastern Algeria: Sétif, Jijel, Constantine, Oum El Bouaghi, Biskra and Ouargla	41 cysts: 24 cattle, 13 sheep and 4 dromedary cysts	*cox*1 sequencing following FTA-card sampling	*E. granulosus* s.s. G1 was predominant; G3 was identified in four cattle cysts	Benchikh El Fegoun *et al.* [5]
Humans, sheep and cattle	Sétif Province, northeastern Algeria	41 isolates: 7 human, 30 sheep and 4 cattle isolates	Partial mitochondrial *cox*1	All isolates belonged to *E. granulosus* s.s.; 19 haplotypes were identified	Kheninef *et al.* [27]
Humans	Mainly northern and central Algeria; one patient from Tindouf	31 cysts	12S-*rrn*S, *nad*5, *nad*2 and qPCR	*E. granulosus* s.s. genotypes G1 and G3 and *E. canadensis* genotype G6/G7	Present study

Sequence comparison with the GenBank database identified 28 isolates as *E. granulosus* s.s. and one isolate as *E. canadensis*. Among the *E. granulosus* s.s. isolates, 27 were assigned to genotype G1 and one isolate (A19) to genotype G3 (Table 3). The 254-bp 12S-*rrnS* sequence obtained from sample A19 differed by two substitutions from that of the published *E. granulosus* s.s. G1 genome (GenBank accession No. AB786664) at two nucleotide positions: T → G at position 12,270 and A → G at position 12,309, and differed from six 12S sequences from CE lesions genotyped as genotype G3 from the routine diagnosis made in the NRC-E. The *nad5* sequence analysis further supported the assignment of A19 to genotype G3, revealing six substitutions over the 683-bp aligned sequence (positions on reference AB786664 (G1/G3): 758 (G → C), 781 (A → G), 1035 (C → T), 1123 (G → A), 1371 (A → G), and 1380 (G → A) as described by Kinkar *et al.* in 2018). Sample A08 was identified as *E. canadensis* G6/G7 based on 12S-*rrn*S sequencing and differed by 30 substitutions over the 240-bp aligned 12S-*rrn*S sequence. Analysis of the *nad*2 sequence revealed substitutions characteristic of genotype G6 according to Laurimäe *et al.* [36]. However, amplification of the *nad*5 fragment was unsuccessful. Therefore, this isolate was conservatively reported as *E. canadensis* G6/G7.

Real-time PCR detected *Echinococcus* DNA in 30/31 samples (96.8%). Twenty-nine samples were positive with the *E. granulosus* s.s. G1/G3 assay, whereas sample A08 was positive with the *E. canadensis* G6/G7 assay, with identical Cq values of 29.2 in both replicates (Table 3). Among the 28 samples positive in both G1/G3 qPCR replicates, the mean Cq value was 28.8, with mean sample values ranging from approximately 22.0 to 43.8. Sample A12, which was negative by conventional 12S-*rrnS* PCR, showed late qPCR amplification in one replicate (Cq = 43.5), while the second replicate remained negative (>45 cycles). According to the predefined positivity criterion of at least one replicate with a Cq value <45, A12 was considered qPCR-positive. However, because no sequencing data were obtained, this sample could not be assigned to a genotype.

No complete PCR inhibition was detected. Two samples (A9 and A16) showed slight PCR inhibition, with Alea Cq values of 35.4 and 35.3 cycles, respectively, although *Echinococcus* spp. DNA was successfully detected in both samples. All remaining samples showed Alea Cq values below 35 cycles (Table 3).

Clinical and epidemiologic characteristics of the molecularly characterized cases are summarized in Table 3. Among the 28 *E. granulosus* s.s. isolates, 17 were obtained from pulmonary cysts, six from hepatic cysts, and five from patients with concomitant pulmonary and hepatic cysts. The single *E. canadensis* genotype G6/G7 isolate was obtained from a hepatic cyst. Patients’ occupations are reported descriptively in Table 3. The *E. granulosus* s.s. genotype G3 isolate was identified in a sample from a production worker, whereas the G6/G7 isolate was identified in a sample from a nurse. Most patients originated from northern and central Algeria, while the single G6/G7 isolate was obtained from a patient originating from Tindouf in southern Algeria.

## Discussion

In Algeria, molecular data on human isolates of *E. granulosus* s.l. remain limited, whereas abattoir-based studies have provided better characterization of genotypic diversity in animals. In this context, our results provide new data on patients in Algeria and offer the opportunity to compare them with regional and international findings to place the identified genotypes within the broader epidemiologic context of human cystic echinococcosis. Our study sheds new light on the genotypes of *E. granulosus* s.l. infecting Algerian patients, showing that *E. granulosus* s.s. genotype G1 was the most frequently identified genotype among the successfully characterized isolates. Among the 29 molecularly characterized samples, 27 were identified as *E. granulosus* s.s. genotype G1, one as genotype G3, and one as *E. canadensis* genotype G6/G7.

At the global level, Alvarez Rojas *et al.* [2] reported that *E. granulosus* s.s. genotype G1/G3 and the *E. canadensis* genotype G6/G7 group accounted for approximately 88.4% and 11.1% of molecularly characterized human isolates, respectively. Together, these genotype groups represent approximately 99.5% of reported human CE cases worldwide [2]. Thus, the genotype distribution observed in the present study, characterized by a marked predominance of *E. granulosus* s.s. and the additional detection of *E. canadensis* is consistent with the global pattern reported for human cystic echinococcosis.

The predominance of *E. granulosus* s.s. among the molecularly characterized human isolates in the present study is consistent with previous Algerian studies reporting its circulation in humans and livestock, including sheep and cattle [55] and is consistent with the recognized importance of domestic transmission involving dogs and livestock in Algeria. However, the present hospital-based study was not designed to quantify the relative contribution of this cycle at the national level. This pattern is also consistent with regional studies reporting the predominance of genotype G1 in human and sheep isolates from Algeria, Morocco, and Tunisia [20, 42, 47].

Additionally, *E. canadensis* genotype G6, known as the “camel strain” [44], contributes less frequently to human CE than G1/G3, but remains epidemiologically important in camel-rearing regions. Dehghani *et al.* [13] reported substantial 12S-rRNA genetic diversity among camel-derived G6/G7 isolates from the Middle East and North and sub-Saharan Africa, suggesting geographically structured patterns of camel-associated transmission. Previous Algerian studies have reported G6-associated isolates in dromedaries from Saharan regions, including Tindouf and Illizi, with several distinct haplotypes suggesting local genetic diversity, as well as occasional human infections, such as the Tuareg case reported in Tamanrasset [44, 55]. The strong association of *E. canadensis* genotype G6 with dromedaries supports its specific epidemiologic role in desert regions [4]. The identification of an *E. canadensis* G6/G7 isolate in a patient from Tindouf is therefore consistent with these previous findings and the importance of dromedary husbandry in this region, highlighting the relevance of camel-associated transmission in the regional epidemiology of CE. However, the precise source of infection in this patient could not be determined.

Lastly, *E. granulosus s.s.* genotype G3, historically referred to as the “buffalo strain”, has been reported sporadically in Algeria, Morocco, and Tunisia, and its contribution to human CE in North Africa appears to be substantially lower than that of genotype G1 [47, 55]. In neighboring Tunisia and Morocco, the G1 genotype is predominant in humans and sheep, while *E. canadensis* is mainly associated with dromedaries [9, 47]. Overall, these regional data indicate the co-circulation of distinct transmission patterns, with a widespread dog–sheep cycle associated with *E. granulosus* s.s. genotype G1, and a camel-associated cycle involving *E. canadensis* genotype G6 in Saharan and camel-rearing regions.

Pulmonary and hepatic cyst localizations were observed in the present series. However, their relative frequencies should be interpreted cautiously because of the hospital-based recruitment strategy, particularly the recruitment of patients through a thoracic surgery department. Previous Algerian studies have also reported both pulmonary and hepatic CE localizations [1, 23]. Slaughterhouse-based studies indicate various occurrence frequencies depending on the species: in sheep, a higher prevalence in the lungs (24.4%) compared to the liver (11.7%) was observed [1]. In humans, proteomic analyses of hydatid fluid have revealed variations related to anatomical locations (liver, lung, vertebra) and suggest the natural tropism of the parasite [56]. Detailed studies on liver lesions have shown a predilection for liver segment VII, often associated with extrahepatic dissemination [17].

Comparative data indicate that CE prevalence is higher in North Africa than in Mediterranean Europe, partly because of practices such as home slaughtering, feeding dogs with infected offal, and the limited use of deworming treatments [41]. An Africa-wide meta-analysis demonstrated a strong association between canine and human infection, with dog infection rates reaching up to 25.6% in North Africa [26]. In Tunisia, despite decades of control efforts, CE remains highly prevalent, highlighting the limitations of approaches based solely on dog treatment or meat inspection [18]. The persistence of CE in Algeria therefore emphasizes the need for an integrated control strategy combining regular praziquantel treatment of dogs, stray-dog management, safe disposal of infected offal, regulation of informal slaughtering, and EG95 vaccination of sheep [3, 12, 35]. In this context, molecular genotyping can contribute to epidemiologic surveillance by identifying circulating *Echinococcus* species and genotypes and by helping to clarify local transmission cycles. This type of information may support prevention and control measures tailored to the dominant intermediate hosts and parasite transmission patterns in different endemic settings [48, 51]. However, most molecular studies conducted in Algeria have focused on livestock samples collected in abattoirs. Although these investigations provide valuable information on parasite diversity in animals, the limited number of molecularly characterized human cases prevents a reliable assessment of the national distribution of *Echinococcus* species and genotypes [20, 34, 44].

Molecular characterization of *E. granulosus* s.l. currently relies on complementary PCR, qPCR, and sequencing approaches [32]. In the present study, qPCR showed a higher detection rate (30/31, 96.8%) than conventional 12S-*rrnS* PCR (29/31, 93.5%). In addition, qPCR provided results within a few hours, whereas conventional PCR followed by sequential analyses required approximately 2–3 days. For samples characterized by both approaches, qPCR species-group assignments were concordant with the corresponding sequencing results. However, genotype-level identification required sequencing of more discriminative mitochondrial markers, further supporting the reliability of qPCR as a rapid and robust molecular tool. Among molecular targets, mitochondrial markers have proven to be the most effective. For initial species identification, the 12S-*rrnS* gene is commonly used as a broad molecular target within the *E. granulosus* s.l. complex [15, 19]. In contrast, *nad5* and *nad2* gene markers represent more discriminative targets, enabling fine differentiation among *E. granulosus* genotypes G1, G3, and *E. canadensis* genotypes G6/G7 [11, 29, 36, 44, 46]. However, poor DNA quality may prevent the amplification of long PCR fragments, potentially explaining the failure to amplify the *nad*2 and *nad*5 gene targets in the present study. Moreover, multilocus approaches combining several mitochondrial markers have been shown to enhance genetic resolution and improve the accuracy of genotyping [40].

The reliability of genotyping, however, depends strongly on the biological matrix analyzed. Hydatid fluid, which is rich in protoscoleces and membranous debris, is readily accessible, but its DNA yield varies according to cyst fertility [24]. In fertile cysts, protoscoleces recovered from hydatid fluid provide suitable parasite material for molecular analysis, whereas germinal-layer tissue can provide parasite DNA when protoscoleces are absent, as it contains parasite cells and therefore provides high mitochondrial DNA concentrations [45]. Algerian studies have further demonstrated that its collection on FTA cards (Fast Technology for Analysis) ensures excellent preservation and robust results, even under abattoir conditions [5, 8]. The laminated layer was long considered of limited value due to its acellular structure [50]. However, parasite DNA was successfully amplified from some laminated-layer preparations, suggesting that these preparations may constitute complementary material when other cyst-derived specimens are unavailable. All these observations are consistent with experimental validations, which highlight the advantage of internal matrices (hydatid fluid and germinal layer) for molecular analyses, while recognizing the laminated layer as a complementary resource under specific circumstances [21, 25, 30].

This study has several limitations. First, the sample size was limited to 31 surgically confirmed cases recruited from two referral hospitals in Algiers, which may have introduced referral and selection bias. Second, most patients originated from northern and central Algeria; therefore, the present findings cannot be considered representative of the national epidemiologic situation and cannot be used to estimate nationwide species or genotype frequencies. Third, some clinical and epidemiologic data, including detailed cyst characteristics, rural or urban residence, history of contact with dogs or livestock, animal-product handling, and travel history were not systematically available for all patients. Therefore, associations between these variables and parasite species or genotypes could not be assessed. Finally, only one specimen type from one cyst was analyzed per patient. Consequently, the relative molecular performance of hydatid fluid, cyst-wall material, and laminated-layer preparations could not be assessed through paired comparisons.

## Conclusion

This study provided molecular characterization of *E. granulosus* s.l. isolates from Algerian patients, highlighting the predominance of *E. granulosus* s.s. genotype G1, together with less frequent detection of genotype G3 and *E. canadensis* genotype G6/G7. Parasite DNA was successfully amplified from several cyst-derived materials, including hydatid fluid, cyst-wall material, and laminated-layer preparations. Although no direct comparison between these matrices was possible, successful amplification from some laminated-layer preparations suggests that this material may represent a complementary option when other cyst-derived specimens are unavailable. The qPCR technique provided rapid and practical detection and species identification, whereas conventional PCR followed by Sanger sequencing remained necessary for detailed genotype characterization. Thus, the choice of molecular approach should depend on the objective: qPCR for rapid detection and diagnostic support, and sequencing-based approaches for higher-resolution genotyping and epidemiologic investigations. Overall, these findings add to current knowledge of human CE molecular epidemiology in Algeria and support the need for broader, geographically representative One Health studies.
